# The Interplay between Transcriptional Factors and MicroRNAs as an Important Factor for Th17/Treg Balance in RA Patients

**DOI:** 10.3390/ijms21197169

**Published:** 2020-09-28

**Authors:** Tomasz Kmiołek, Ewa Rzeszotarska, Anna Wajda, Ewa Walczuk, Ewa Kuca-Warnawin, Katarzyna Romanowska-Próchnicka, Barbara Stypinska, Dominik Majewski, Pawel Piotr Jagodzinski, Andrzej Pawlik, Agnieszka Paradowska-Gorycka

**Affiliations:** 1 Department of Molecular Biology, National Institute of Geriatrics, Rheumatology and Rehabilitation, 02-637 Warsaw, Poland; tomasz.kmiolek@spartanska.pl (T.K.); annawajda2046@gmail.com (A.W.); ewa.walczuk@spartanska.pl (E.W.); barbara.stypinska@wp.pl (B.S.); paradowska_aga@interia.pl (A.P.-G.); 2 Department of Pathophysiology and Immunology, National Institute of Geriatrics, Rheumatology and Rehabilitation, 02-637 Warsaw, Poland; ewa.kuca-warnawin@spartanska.pl; 3 Department of Connective Tissue Diseases, National Institute of Geriatrics, Rheumatology and Rehabilitation, 02-637 Warsaw, Poland; katarzyna.prochnicka@gmail.com; 4 Department of Pathophysiology, Warsaw Medical University, 02-091 Warsaw, Poland; 5 Department of Rheumatology and Internal Medicine, Poznan University of Medical Science, 61-701 Poznan, Poland; dmajes@poczta.onet.pl; 6 Department of Biochemistry and Molecular Biology, Poznan University of Medical Sciences, 61-701 Poznan, Poland; pjagodzi@ump.edu.pl; 7 Department of Physiology, Pomeranian Medical University, 70-204 Szczecin, Poland; pawand@poczta.onet.pl

**Keywords:** rheumatoid arthritis (RA), Treg, Th17, gene expression, microRNA, transcriptional factor

## Abstract

MicroRNAs regulate gene expression of transcriptional factors, which influence Th17/Treg (regulatory T cells) balance, establishing the molecular mechanism of genetic and epigenetic regulation of Treg and Th17 cells is crucial for understanding rheumatoid arthritis (RA) pathogenesis. The study goal was to understand the potential impact of the selected microRNAs expression profiles on Treg/Th17 cells frequency, RA phenotype, the expression profile of selected microRNAs, and their correlation with the expression profiles of selected transcriptional factors: SOCS1, SMAD3, SMAD4, STAT3, STAT5 in RA; we used osteoarthritis (OA) and healthy controls (HCs) as controls. The study was conducted on 14 RA and 11 OA patients, and 15 HCs. Treg/Th17 frequency was established by flow cytometry. Gene expression analysis was estimated by qPCR. We noticed correlations in RA Th17 cells between miR-26 and SMAD3, STAT3, SOCS1; and miR-155 and STAT3—and in RA Treg cells between miR-26 and SOCS1; miR-31, -155 and SMAD3; and miR-155 and SMAD4. In RA Tregs, we found a negative correlation between miR-26, -126 and STAT5a. The expression level of miR-31 in Th17 cells from RA patients with DAS28 ≤ 5.1 is higher and that for miR-24 is greater in Tregs from patients with DAS28 > 5.1. MiR-146a in Tregs is higher in rheumatoid factor (RF) positive RA patients.

## 1. Introduction

Rheumatoid arthritis (RA) is one of the most widespread inflammatory diseases that affect the immune system. The most common symptoms are pain, joint swelling, and disability. In RA occurs proliferative synovitis leading to bone and cartilage destruction. The inflamed synovium contains synovial fibroblasts, macrophages, and T cells that secrete inflammatory cytokines. They activate osteoclasts, causing bone destruction. In RA occurs undue immune response of T cells. CD4+ T cells contain helper T cells (Th cells) and they stimulate the immune responses as well as regulatory T cells (Treg cells) that control these responses. Th cells include Th1, Th2, and Th17 cells subsets. RA is characterized by an imbalance in Th17/Treg, and Th17 is more activated than Treg [1].

In osteoarthritis (OA), disease joint surfaces are damaged, resulting in a lack of maintaining joint cartilage homeostasis. Damages are caused by excessive mechanical stress like tensile strains and compression forces, trauma, or inflammation. It results in structural damage and deformation [2].

In recent times, microRNAs are considered as an essential player connected with the onset as well as the progression of osteoarthritis [1,3,4]. MicroRNAs (miRNAs, miRs) are single-stranded, conserved, noncoding RNAs, which have approximately 22 nucleotides in length. There has been a lot of interest in miRNAs research in recent years, and more than two thousand microRNAs have been discovered so far. It is becoming increasingly clear that miRNAs contribute to the number of biological processes, e.g., apoptosis and cell proliferation. MicroRNAs regulate gene expression post-transcriptionally. About one-third of the genes in the human genome is under the control of the miRNAs [5]. Apart from the repression of translation, miRNAs moreover begin the affinity and enrollment of mRNA restriction factors, which leads to degradation of mRNA and disrupts gene expression. MicroRNAs regulate both acquired and innate immunity by their contribution to the cytogenesis and generation of immune cells (e.g., dendritic cells, T cells, B cells). Altered miRNAs stimulate the production of undue autoantibodies and inflammatory cytokines secretion. The abovementioned processes lead to an imbalance of the immune system. As a result, miRNAs are linked with various autoimmune diseases. MiRNAs have a crucial role in the pathogenesis of diseases like rheumatoid arthritis (RA), systemic lupus erythematosus (SLE), multiple sclerosis (MS), and primary biliary cholangitis (PBC) [4,5].

Yang et al. suggested that elevated miR-126 expression induces the hypomethylation of CD70 and CD11a genes, by the depression of DNMT1 protein, causing the onset and progression of RA [6]. Altered miR-24 occurs in RA and upregulation of this miRNA regulating the production of cytokines and causing deterioration of arthritis [7,8]. MiR-26a and miR-155 are linked to the immune responses and can suppress immune cell apoptosis; increase the activity of the immune system; and cause specific organ damage, including joint, skin, kidney, and lung [8]. Numerous microRNAs have been reported to be connected with synovial cell proliferation, osteoclast differentiation, and inflammatory cytokines and they have the potential to be used in the treatment of RA. MiR-155 and miR-146a are mentioned as representative miRNAs associated with the RA condition [2]. MiR-146a is critical for Treg cells suppressor function. The insufficiency of miR-146a in regulatory T cells caused a breach of immunological tolerance [9]. Suppressor of cytokine signaling 1 (SOCS1), the key negative controller of Janus kinase/Signal transducers and activators of transcription (JAK/STAT) signaling pathway [10], has been reported as the main target of miR-155 in peripheral blood mononuclear cells (PBMCs) of RA patients [2,11]. MiR-155 suppresses the expression of SOCS1 in activated CD4+ T cells and this leads to the stimulation of Interleukin-6/Signal transducer and activator of transcription 3 (IL-6/STAT3), and Interleukin-2/Signal transducer and activator of transcription 5 (IL-2/STAT5) signaling pathways, as well as the initiation of Treg/Th17 cells functions and differentiation [10].

Since microRNAs regulate gene expression of transcriptional factors, which impact Th17/Treg balance, assignment of the molecular mechanism of epigenetic as well as genetic regulation of Th17 and Treg cells is vital for comprehending RA etiology and pathogenesis. This study aimed to understand the potential influence of the selected microRNAs’ expression profiles on the phenotype of RA. Moreover, we wanted to study and describe the regulation of five selected transcriptional factors, SOCS1, SMAD3, SMAD4, STAT3, and STAT5, by carefully chosen microRNAs in RA and control groups: OA and healthy control (HC). Moreover, we search for microRNAs that could be RA biomarkers.

## 2. Results

### 2.1. Increased Expression Levels of miR-146a and miR-155 in Treg Cells from RA and OA

**Figure 1:**
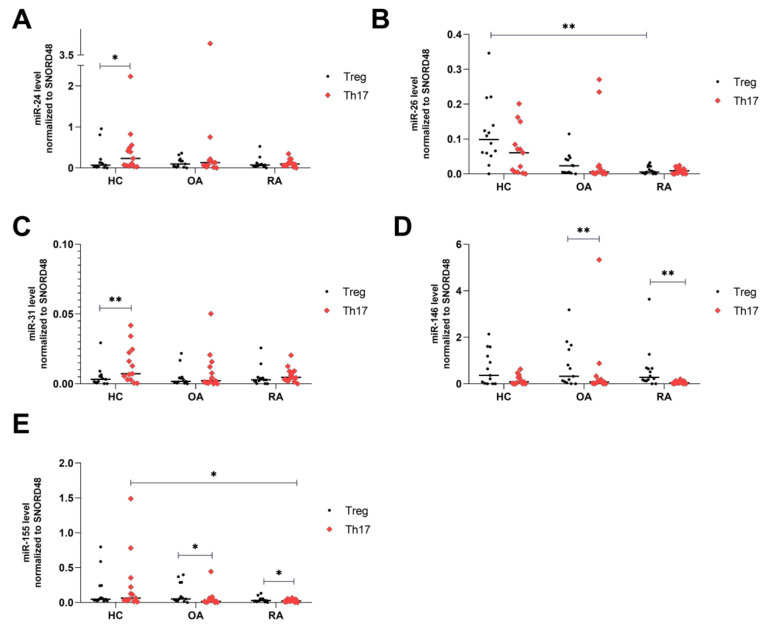
MicroRNA comparison in Treg and Th17 in healthy controls (HCs), osteoarthritis (OA), and rheumatoid arthritis (RA). MiR-24 level normalized to SNORD48 (**A**), miR-26 level normalized to SNORD48 (**B**), miR-31 level normalized to SNORD48 (**C**), miR-146a level normalized to SNORD48 (**D**), miR-155 level normalized to SNORD48 (**E**). Data are presented with the median as the scatterplot graph. Subtypes of Treg vs. Th17 cells within groups were analyzed by the Wilcoxon test. Multiple comparisons of HCs vs. RA vs. OA were conducted with the Kruskal–Wallis test with Dunn’s post hoc. HC (n = 15), OA (n = 11), RA (n = 14). * p < 0.05, ** p < 0.01.

Analysis of miRNAs expression levels in freshly isolated Th17 and Treg cells (Table S1) have shown that expression of miR-24 was 3 times higher in Th17 cells than in Treg cells from healthy subjects (p = 0.04). We did not see noticeable differences in OA and RA patients in the miR-24 expression level between Treg and Th17 cells (Figure 1A). We observed higher miR-26 expression levels in Treg cells from HCs than in RA Treg cells (p = 0.014). There were no differences in the miR-26 expression levels between Th17 cells and Treg cells in RA and OA patients (Figure 1B). We also observed that the miR-31 expression was 2 times higher in Th17 cells than in Treg cells from HCs (p = 0.0046, Figure 1C). For miR-146a, we noticed that its expression was 4 times higher in OA Treg cells than in OA Th17 cells (p = 0.008, Figure 1D), and 6 times higher in RA Treg cells than in RA Th17 cells (p = 0.0031, Figure 1D). MiR-155 expression was significantly higher in RA and OA Treg cells than in RA and OA Th17 cells (both p = 0.04, Figure 1E). In contrast, miR-155 expression was significantly higher in HC Th17 cells than in RA Th17 cells (p = 0.04, Figure 1E).

**Figure 2:**
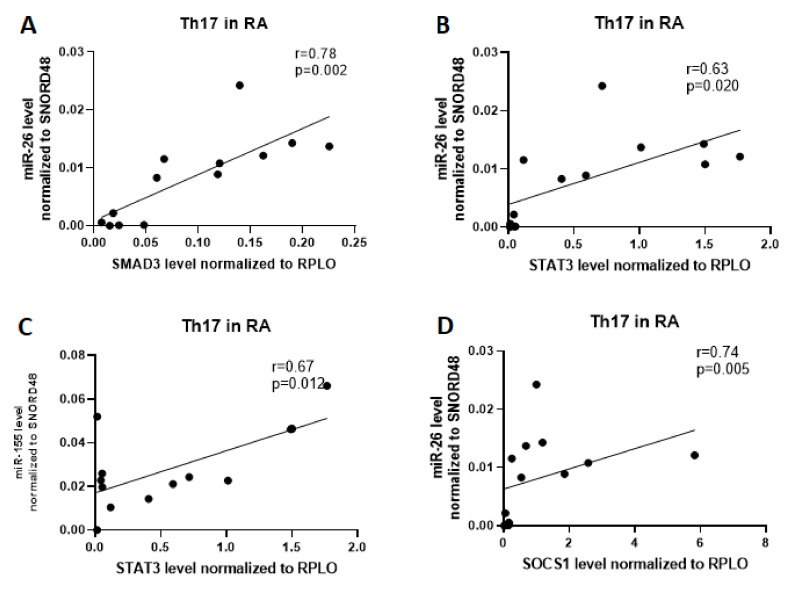
Correlation analysis of microRNA to SMAD3, STAT3, and SOCS1 in RA patients in Th17 cells. (**A**)- correlation between SMAD3 and miR-26; (**B**) correlation between STAT3 and miR-26; (**C**) correlation between STAT3 and miR-155; (**D**) correlation between SOCS1 and miR-26.

In the next step, we examined whether there exists a relationship between the mRNA expression and miRNAs expression. For this purpose, we analyzed correlations between examined miRNA and mRNA in the subpopulation of Th17 and Treg cells in RA patients. We found that the expression of miR-26 was positively correlated with SMAD3 (r = 0.78, p = 0.002), STAT3 (r = 0.63, p=0.02), and SOCS1 (r = 0.74, p = 0.005) expression, and expression of miR-155 was positively correlated with STAT3 expression (r = 0.67, p = 0.012) in Th17 cells from patients with RA (Figure 2).

**Figure 3:**
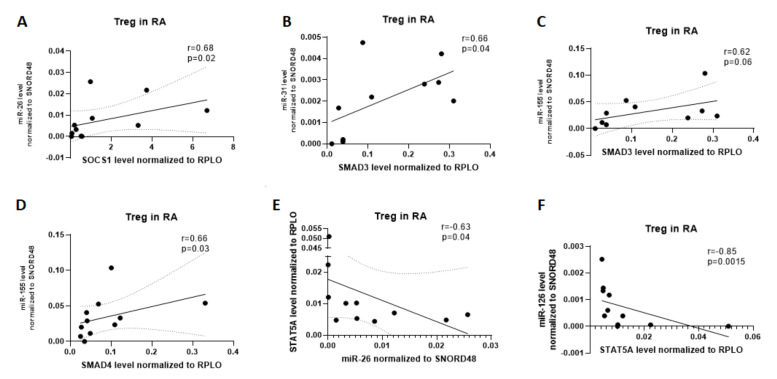
Correlation analysis of microRNAs to SMAD3, SMAD4, SOCS1, and STAT5A in RA patients in Treg cells. (**A**) correlation between SOCS1 and miR-26; (**B**) correlation between SMAD3 and miR-31; (**C**) correlation between SMAD3 and miR-155; (**D**) correlation between SMAD4 and miR-155; (**E**) correlation between miR-26 and STAT5a; (**F**) correlation between STAT5a and miR-126.

In contrast, in RA Treg cells, a strong positive correlation was observed between miR-26 and SOCS1 (r = 0.68, p = 0.02), between miR-31 and SMAD3 (r = 0.66, p = 0.04), and between miR-155 and SMAD3 (r = 0.62, p = 0.06) and SMAD4 (r = 0.66, p = 0.04). Furthermore, a significant, but negative correlation was observed between miR-26 and STAT5A (r = −0.63, p = 0.04) and between miR-126 and STAT5A (r = −0.85, p = 0.0015; Figure 3).

### 2.2. Lack of Correlation between microRNA and mRNA in Th17 Cells in Control Groups: OA Patients and Healthy Subjects

**Figure 4:**
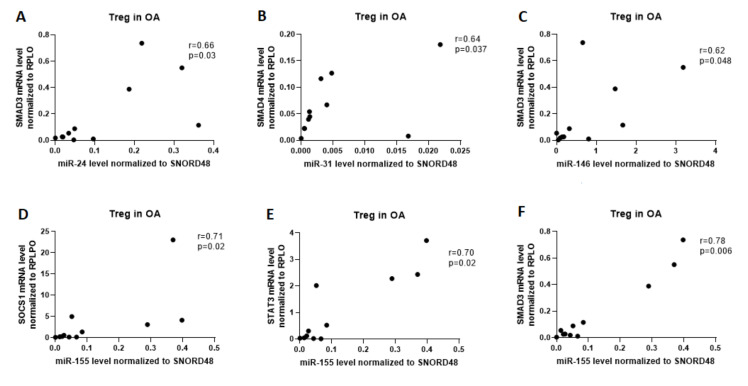
Correlation analysis of microRNAs to SMAD3, SMAD4, SOCS1, and STAT3 in OA patients in Treg cells. (**A**) correlation between miR-24 and SMAD3; (**B**) correlation between miR-31 and SMAD4; (**C**) correlation between miR-146a and SMAD3; (**D**) correlation between miR-155 and SOCS1; (**E**) correlation between miR-155 and STAT3; (**F**) correlation between miR-155 and SMAD3.

The aberrant expression of microRNAs through affecting the expression of target genes involved in Th17/Treg differentiation/function may contribute to the inflammatory responses in RA patients. To better understand the role of the examined miRNAs in Treg/Th17 cells balance, we conducted a correlation analysis between studied miRNA and expression of transcriptional factors playing a central role in the development of T cells. Data indicated that in OA Treg cells, a strong positive correlation was observed between miR-24 and SMAD3 (r = 0.66, p = 0.03), miR-31 and SMAD4 (r = 0.64, p = 0.037), miR-146a and SMAD3 (r = 0.62, p = 0.048), as well as between miR-155 and SOCS1 (r = 0.71, p = 0.02), STAT3 (r = 0.70, p = 0.02) and SMAD3 (r = 0.78, p = 0.006; Figure 4). In OA Th17 cells, we did not observe the correlation between examined miRNA and mRNA.

**Figure 5:**
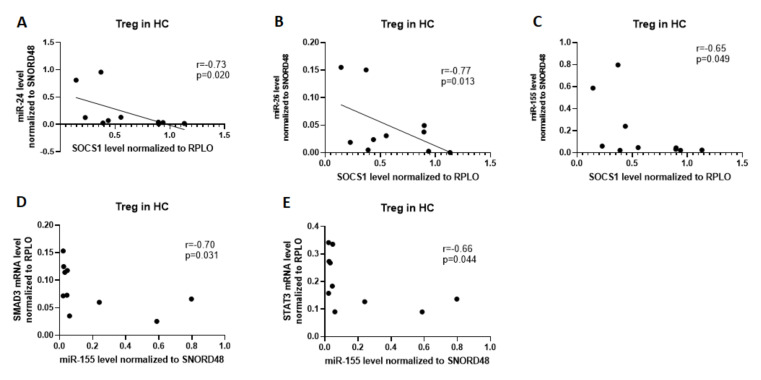
Correlation analysis of microRNAs to SMAD3, SOCS1, and STAT3 in HCs in Treg cells. (**A**) correlation between SOCS1 and miR-24; (**B**) correlation between SOCS1 and miR-26; (**C**) correlation between SOCS1 and miR-155; (**D**) correlation between miR-155 and SMAD3; (**E**) correlation between miR-155 and STAT3.

We found that the expression of miR-24 was positively correlated with SOCS1 (r = 0.73, p = 0.02), expression of miR-126 with SOCS1 (r = 0.77, p = 0.013), and expression of miR-155 with SOCS1 (r = 0.65, p = 0.049) in Treg cells from healthy subjects. In Treg cells from healthy subjects, we observed a high, negative correlation between miR-155 and SMAD3 (r = 0.70, p = 0.031) and STAT3 (r = 0.66, p = 0.044; Figure 5). In HCs’ Th17 cells, we also did not observe the correlation between examined miRNA and mRNA.

### 2.3. Correlation Analysis Of Related Target Genes Based on the mRNA–miRNA Interaction Network

**Figure 6:**
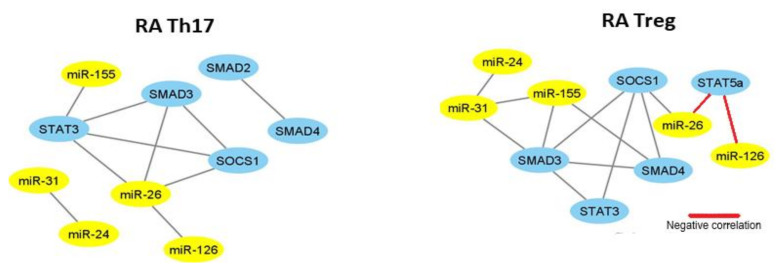
The network of high, significant correlation between selected microRNAs and transcriptional factors in RA patients (n = 14) in Th17 and Treg cells constructed in Cytoscape (coefficient of correlation higher than 0.6, significance < 0.05).

Subsequently, we established miRNA–mRNA coexpression network analysis to identify the possible modulating mechanisms of the mRNAs. The miRNA–mRNA coexpression network analysis was constructed in Cytoscape software. This coexpression network analysis revealed that in RA Th17 cells, expression of miR-126 is correlated with expression of miR-26 and both may regulate expression of STAT3, SMAD3, and SOCS1 (Figure 6). In RA Treg cells, we did not observe this relationship.

**Figure 7:**
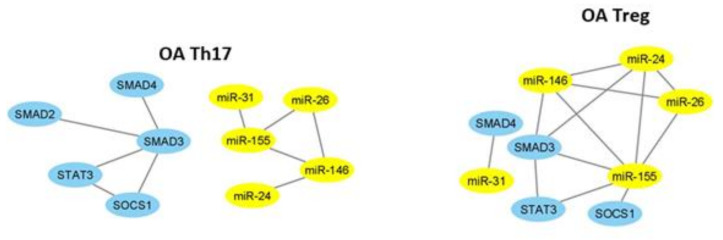
The network of high, significant correlation between selected microRNAs and transcriptional factors in OA patients (n = 11) in Th17 and Treg cells constructed in Cytoscape (coefficient of correlation higher than 0.6, significance < 0.05).

**Figure 8:**
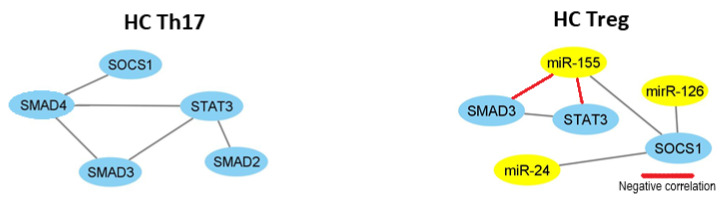
The network of high, significant correlation between selected microRNAs and transcriptional factors in HC (n = 15) in Th17 and Treg cells constructed in Cytoscape (coefficient of correlation higher than 0.6, significance < 0.05).

As it is shown in correlation networks (Figure 7 and Figure 8), we did not find any correlation between miRNA and mRNA in Th17 cells from OA patients and healthy subjects. In OA Treg, we were able to observe correlation mostly between miR-155 and STAT3, SOCS1, and SMAD3. Moreover, SMAD3 was also correlated with miR-146a and miR-24. Additionally, we noticed a correlation between miR-31 and SMAD4.

Similar to RA and OA patients, we prepared a correlation network to show how tested microRNAs may affect mRNAs in our healthy controls. We did not find any regulation of microRNA and mRNA in Th17 cells from healthy subjects. On the other hand, in Treg cells from healthy subjects, we observed that miR-126 and miR-24 were positively correlated with SOCS1 and that miR-155 was negatively correlated with SMAD3 and STAT3, but positively with SOCS1 (Figure 8).

### 2.4. MicroRNAs Are Significantly Correlated with Each Other in Th17 and Treg Cells in RA and OA Patients

**Figure 9:**
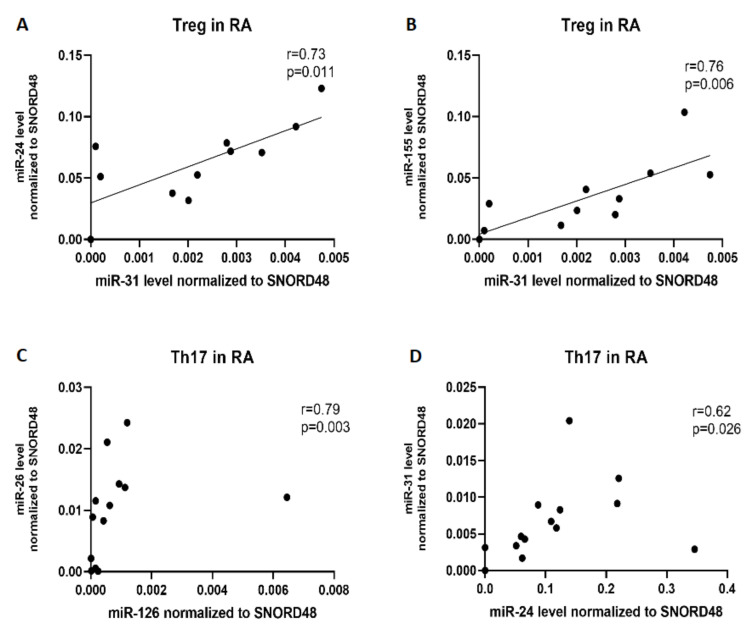
Correlation between microRNAs in Th17 and Treg cells in RA patients. (**A**) correlation between miR-31 and miR-24; (**B**) correlation between miR-31 and miR-155; (**C**) correlation between miR-126 and miR-26; (**D**) correlation between miR-24 and miR-31.

Next, we investigated the correlation between examined miRNAs expression in Th17 and Treg cells isolated from RA and control groups: OA patients as well as from healthy subjects. We observed significant, positive correlations between miR-31 and miR-24 (r = 0.73, p = 0.011), between miR-31 and miR-155 (r = 0.76, p = 0.006) in Treg from RA, as well as between miR-126 and miR-26 (r = 0.79, p = 0.003) and between miR-24 and miR-31 (r = 0.62, p = 0.0026) in Th17 cells from RA patients (Figure 9).

Figure S1 presents correlations between miRNAs in Treg cells from OA patients. We noticed a significant, positive correlations between miR-155 and miR-24 (r = 0.81, p = 0.001), between miR-155 and miR-26 (r = 0.75, p = 0.004), and between miR-26 and miR-24 (r = 0.62, p = 0.02).

Whereas in OA Th17 cells (Table S2), the highest correlation has been observed between expression of miR-24 and miR-146a (r = 0.70, p = 0.0006), expression of miR-26 and miR-146a (r = 0.83, p < 0.0001), expression of miR-26 and miR-155 (r = 0.89, p < 0.0001); and also between expression of miR-31 and miR-155 (r = 0.70, p = 0.007), and expression of miR-146a and miR-155 (r = 0.73, p = 0.004). In healthy controls, we have not noticed correlations between examined microRNAs in Th17 and Treg cells.

### 2.5. DAS-28, Anti-CCP and Rheumatoid Factor (RF) Parameters in Relation to miR-24, -26, -31, -146a, and -155 Expression Levels in Th17 and Treg Cells from RA Patients

To assess the contribution of the examined miRNAs in the RA phenotype, we stratified our patients based on the disease activity, anti-CCP presence, and RF presence. Based on disease activity, we divided RA patients into patients with active disease with DAS-28 > 5.1 (n = 6) and patients with moderate or low disease activity with DAS-28 ≤ 5.1 (n = 8).

**Figure 10:**
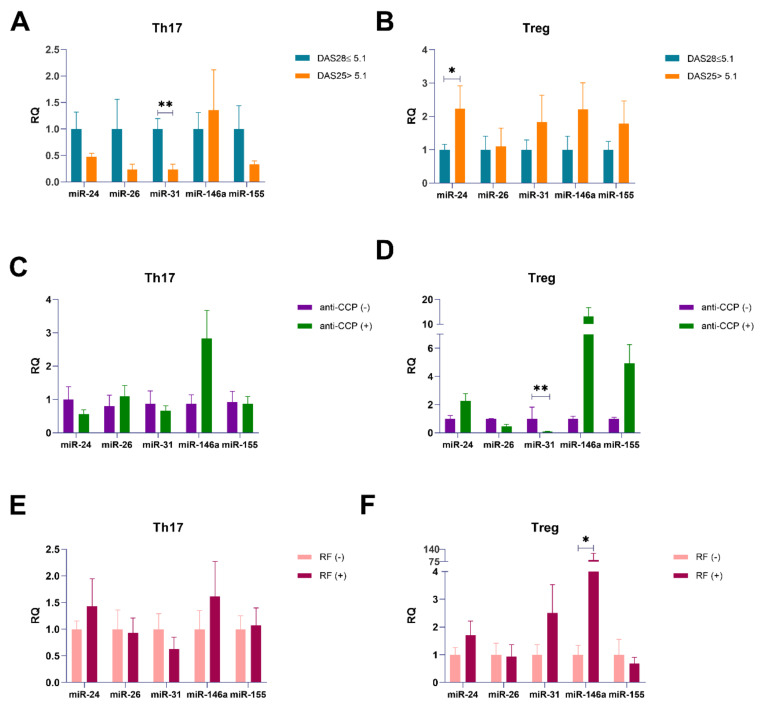
DAS-28 (**A**,**B**), anti-CCP (**C**,**D**), and rheumatoid factor (RF) (**E**,**F**) parameters in relation to miR-24, miR-26 miR-31, miR-146a, miR-155 expression levels in Th17 (**A**,**C**,**E**) and Treg (**B**,**D**,**F**) cells from RA patients. Data are presented as mean ± SEM. Expression of patients with DAS28 ≤5.1 (**A**,**B**), negative anti-CCP (**C**,**D**), and negative RF (**E**,**F**) was taken as 1. The Mann–Whitney test was used. * p < 0.05, ** p < 0.01.

MiR-31 expression was higher in RA patients with DAS-28 ≤5.1. We noticed significantly higher expression levels of miR-31 in Th17 cells from RA patients with DAS-28 ≤ 5.1 (p = 0.002). In Treg cells from RA patients with DAS28 > 5.1, miR-24 expression levels is twice higher than in those with DAS28 ≤ 5.1 (p = 0.048). Other outcomes were not statistically significant, however, it is interesting that in RA Treg cells with DAS28 > 5.1, we observed higher expression levels of miR-146a and miR-155 (Figure 10A,B). Moreover, we also found that miR-24, miR-26, miR-31, and miR-155 were downregulated, while miR-146a were upregulated in Th17 cells from RA patients with DAS28 ≤ 5.1.

Downregulation of miR-31 in Treg cells obtained from anti-CCP positive RA patients. Our results demonstrated that in Treg cells from anti-CCP positive RA patients, expression of miR-31 was significantly lower than in anti-CCP negative RA patients (p = 0.006; Figure 10D). In contrast, in Treg cells from anti-CCP positive RA patients, we noticed twice higher expression level for miR-24, 13 times higher for miR-146a, and 5 times higher for miR-155 than in anti-CCP negative RA patients; however, results were not significant. We also noticed that in Treg cells from anti-CCP negative RA patients, expression level of miR-26 is twice more than in anti-CCP positive patients; these outcomes were not significant. We also observed that in Th17 cells from anti-CCP positive RA patients, expression of miR-146a was higher and miR-24 and miR-31 were lower than in anti-CCP negative RA patients; these results were also not significant.

The higher expression level of miR-146a in RA patients with RF. In the present study, we reported almost twice higher miR-24, 2.5 times higher miR-31, and 82 times higher miR-146a (p = 0.02; Figure 10E,F) expression levels in Treg cells from RA patients with RF than in Treg cells from RA patients without RF. We also observed that expression levels of miR-155 were almost two times lower in Treg cells from RA patients with RF than in RA patients without RF, but this difference was not statistically significant. In contrast, expression levels of miR-126 were similar in both RF-positive and RF-negative RA patients. In RA patients with RF, we also observed the upregulation of miR-31 in Treg cells compared to Th17 cells.

MiR-26 and miR-155 May Be Good Potential Biomarkers for RA and OA. To estimate the further potential value of the examined miRNAs individually or as a panel as RA and/or OA biomarkers, receiver operating characteristic (ROC)–AUC (Area Under Curve) analyses were performed. The outcomes are presented in Table 1. MiR-26 after comparison of RA and HCs in Th17 (AUC = 0.75, p = 0.02) and Treg cells (AUC = 0.92, p = 0.0002) showed a prognostic value. We also revealed this potential for miR-26 in OA vs. HCs in Treg cells (AUC = 0.86, p = 0.0013). MiR-155 after comparison of RA and HCs in Th17 (AUC = 0.80, p = 0.006) and Treg cells (AUC = 0.73, p= 0.048) revealed a prognostic value. We also exposed this potential for miR-155 in OA vs. HCs in Th17 cells (AUC = 0.75, p = 0.03).

**Figure 11:**
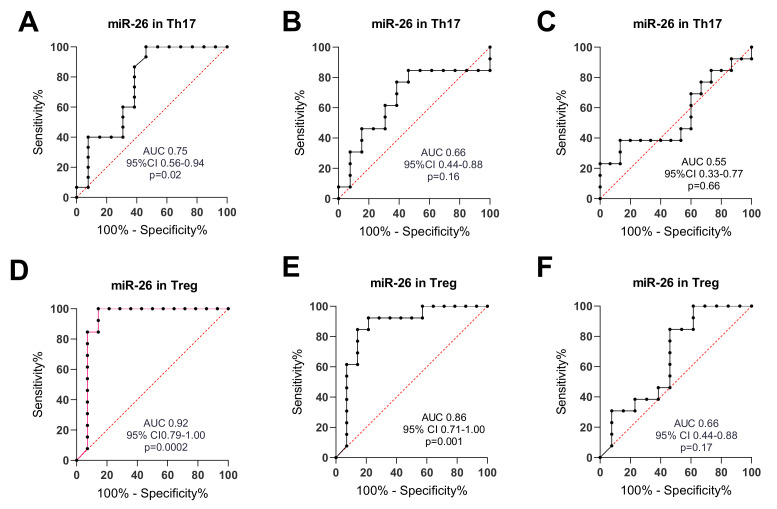
ROC curve analysis for miR-26. (**A**) RA vs. HC in Th17; (**B**) OA vs. HC in Th17; (**C**) RA vs. OA in Th17; (**D**) RA vs. HC in Treg; (**E**) OA vs. HC in Treg; (**F**) RA vs. OA in Treg.

In the next step, we conducted the ROC curve analysis and estimated AUC to assess the diagnostic potential of candidate microRNAs that could distinguish RA and OA patients from healthy controls. Figure 11A–F shows the ROC curve analysis for miR-26 in Th17 and Treg cells in RA, OA, and HCs.

The highest AUC value was obtained for miR-26 discriminating RA from healthy controls (AUC 0.92) and also differentiating OA from HCs (AUC 0.86) in Treg cells. The high AUC value was also obtained for miR-26 distinguishing RA from HCs (AUC 0.75) in Th17 cells.

**Figure 12:**
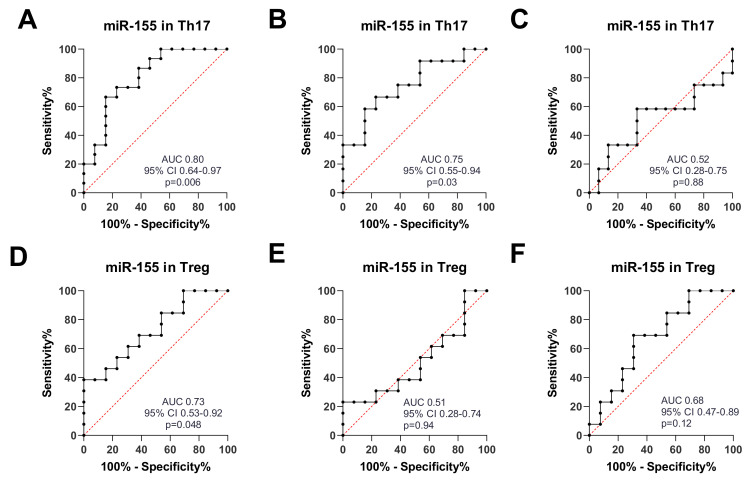
ROC curve analysis for miR-155. (**A**) RA vs. HC in Th17; (**B**) OA vs. HC in Th17; (**C**) RA vs. OA in Th17; (**D**) RA vs. HC in Treg; (**E**) OA vs. HC in Treg; (**F**) RA vs. OA in Treg.

Figure 12A–F shows the ROC curve analysis for miR-155 in Th17 and Treg cells in RA, OA, and HCs. The highest AUC value was obtained for miR-155 discriminating RA from healthy controls in Th17 cells (AUC 0.80) and Treg cells (AUC 0.73) and also differentiating OA from HCs in Th17 cells (AUC 0.75).

Setting a cutoff value at the specific level permitted us to receive the maximum sensitivity and specificity values (Table S3). For miR-26 in RA vs. HC, we obtained 93.33% sensitivity and 53.85% specificity in Th17 (cutoff value = 0.022) and 84.62% sensitivity and 92.86% specificity in Treg cells (cutoff value = 0.025). In OA vs. HC, those values were 76.92% sensitivity and 61.54% specificity (cutoff value = 0.020) in Th17; and 84.62% sensitivity and 78.57% specificity (cutoff value = 0.051) in Treg cells. For miR-155 in RA vs. HC, we set a cutoff values at the levels 0.046 in Th17 (80% sensitivity, 61.54% specificity) and 0.118 in Treg cells (92.31% sensitivity, 30.77% specificity), respectively. For miR-155 in OA vs. HC, the cutoff values were 0.070 in Th17 (83.33% sensitivity, 46.16% specificity) and 0.075 in Treg cells (61.54% sensitivity, 30.77% specificity), respectively.

### 2.6. Multivariable Logistic Regression

Based on the results of the likelihood ratio test (LRT), multivariable logistic regression with a combination of miR-24, miR-26, miR-31, miR-146a, miR-155 as a predictor vector was used to assess their diagnostic accuracy for the possible diagnostic potential. Figure S2 presents ROC analysis for the combination of miR-26 and miR-155 in Th17 RA vs. HCs and miR-146a in Treg OA vs. HCs. Significant diagnostic potential in RA Th17 cells (AUC = 0.811) has only been demonstrated for the combination of miR-26 and miR-155. In Treg from OA patients, only miR-146a has been revealed as a significant estimator in the model (AUC = 0.4196).

## 3. Discussion

In the last years, we observe substantial progress in the field of miRNA-mediated regulation of immune function and T cell development. The clarification of miRNAs role in lymphocytes subtypes development/differentiation can be used to enhance our understanding of the molecular pathways that are involved in immune regulation. Currently, there is limited knowledge about the specific miRNAs participated in the regulation of immune-related molecular processes and to what extent their activity promotes rheumatoid arthritis pathogenesis. Rheumatoid arthritis is an autoimmune disease, which affects joints, and symptoms include pain and lead to disability through cartilage and bone destruction. This study confirmed the major imbalance between Th17 cells and Treg cells in patients with active RA, as was previously reported in the literature [12]. MicroRNAs play a multifaceted role in the development of RA and are part of the complex net of epigenetic interactions. Alternations to these interactions might be results of the disease or a contributing factor in RA pathogenesis [13]. Earlier studies on animal models have also confirmed the role of mir-155 as one of the factors contributing to arthritis development. Results of serum testing in arthritic rats showed an increased level of miR-155; on the other hand, mice lacking miR-155 were resistant to collagen-induced arthritis [14,15]. In the present study, we decided to focus on microRNAs since they are responsible for regulating gene expression of transcriptional factors that impact Th17/Treg balance [2,12]. Understanding the molecular pathway of genetic and epigenetic regulation of Treg and Th17 cells is critical to understand RA etiology and pathogenesis. We are the first, to our knowledge, who analyzed microRNAs from Th17 and Treg cells obtained from RA in comparison to control groups—OA patients also characterized among others by joint damage, but with different pathophysiology and without an autoimmune component and healthy subjects—and correlated them with some transcriptional factors important in Treg and Th17 cells differentiation.

The next focus of our study was to analyze the correlation between microRNAs and mRNAs in Treg and Th17 cells in tested groups used in this study: RA and controls; OA and HCs. In our study, we have shown a correlation between miR-155 and STAT3 as well as between miR-26 and STAT3, SMAD3, and SOCS1 in RA Th17 cells. Che et al. have shown that miR-155 shows the potential to be used as a biomarker [14]. For RA Treg cells, we found a correlation between miR-155 with SMAD3 and SMAD4, which again confirms the possibility to use miR-155 as a biomarker [16,17,18,19]. In the present paper, we have also shown a correlation between miR-31 and SMAD3 as well as between miR-26 and SOCS1; to our knowledge, our study is the first showing these correlations in RA patients. However, the correlation between miR-31 and SMAD3 has already been reported by Hu et al. [20]. In Th17 cells from OA patients and healthy subjects, we did not found a correlation between analyzed microRNAs and mRNAs. On the other hand, our results confirmed earlier studies that in OA Treg cells, there exists/occurs/is a correlation between miR-24 with SMAD3; miR-31 with SMAD4; miR146a with SMAD3; and miR-155 with SOCS1, SMAD3, and STAT3 [17,18,21,22]. In accordance with an earlier study, in Treg cells from healthy subjects, we also found a few correlations between miR-24 and SOCS1, miR-126 and SOCS1, miR-155 and SOCS1; and two negative correlations between miR-155 and SMAD3 and STAT3 [18,19,21]. Both earlier studies and our present study indicate miR-155 as a potential diagnostic biomarker for RA/OA detection, because miR-155 has a negative correlation with SMAD3 and STAT3 in healthy subjects, but positive in RA patients and OA patients.

Analysis of selected microRNAs’ expression in Treg and Th17 cells confirmed differences between Treg and Th17 cells, which is consistent with other studies [13,23]. We observed that the expression of miR-26 is significantly higher in healthy subjects than in RA patients. MiR-31, like miR-24, demonstrated a significant change in expression between Treg and Th17 cells within the HCs group. Our results for miR-146a and miR-155 confirmed the results of previous studies on the expression of microRNAs in RA [23]. We observed that miR-146a expression level in RA and OA Treg was significantly higher than in Th17 cells. The last studies demonstrated that miR-146a plays a considerable role in various aspects of immunopathogenesis. Our results emphasize the role of miR-146a as an anti-inflammatory agent. The downregulating miR-146a expression in Treg cells may promote their proliferation and alleviate the inflammation process. In comparison with the fact that miR-146a preferably reduces the proinflammatory immune response, miR-155 strengthens inflammation, which is consistent with our observations. In the present study, we observed that miR-155 expression in Th17 cells was higher in HCs than in RA patients and that miR-155 expression was higher in Treg cells from RA and OA patients than in Th17 cells. Treg cells from active RA patients with high disease activity (we have 42% RA patients with DAS-28 > 5.1) expressed elevated levels of SOCS1—a target of miR-155—which negatively regulates inflammatory processes. Moreover, our study confirms the value of miR-146a and miR-155 as an important factor in early detection of RA, but we were also able to show the potential use of miR-26 as a biomarker [17,23,24,25].

The present study revealed significant correlations between particular microRNAs. In RA Treg cells, we noticed the correlation between miR-31 and miR-24 as well as between miR-31 and miR-155 levels. In RA Th17 cells, correlations were between miR-126 and miR-26, miR-24, and miR-31 levels. OA Treg cells showed correlations between miR-155 and miR-24, miR-155 and miR-26, miR-26, and miR-24. In OA Th17 cells we saw significant correlations between miR-24 and miR-146a, miR-26 and miR-146a, miR-26 and miR-155, miR-31 and miR-155, and miR-146a and miR-155. In Bae et al. meta-analysis, levels of circulating miR-146a were significantly higher in RA patients than in controls. This study suggests the noteworthy role of miR-146a levels in RA proinflammatory processes [26].

Our study revealed some connections between microRNAs’ expression levels and clinical parameters. We observed in RA patients with DAS28 ≤ 5.1 significantly higher miR-31 expression levels in Th17 cells and contrarily, in RA patients with DAS28 > 5.1 twice higher miR-24 in Treg cells. We also noticed in RA Treg cells upregulation of miR-155 and miR-146a, when DAS28 > 5.1. MiR-26 was at a similar level, independently of DAS score; however, these results were not statistically significant. Li et al. found that in PBMCs from active RA patients, miR-155 expression is positively correlated with DAS28 [11]. Moreover, miRNA-146a in RA patients was also positively correlated with DAS28 [27]. We did not notice significant differences in the anti-CCP level in RA patients, excluding that miR-31 in RA with positive anti-CCP was downregulated in comparison to the level observed in anti-CCP negative RA patients. Treg cells of RA patients with RF-positive results were characterized by a significantly higher level of miR-146a expression.

Our additional analysis revealed that the miR-26 and miR-155 reveal significant diagnostic potential for RA differentiation from healthy subjects. Moreover, high diagnostic potential has been revealed for the combination of miR-155 and miR-26 in RA Th17 cells. MiR-26a was reported as overexpressed in RA PBMCs and plasma in comparison to HC [7,19,28]. During interleukin (IL)-17 differentiation and T CD4+ cells generation, upregulation of miR-26a occurs. T CD4+ cells are crucial in RA pathology [27]. MiR-155 insufficiency reduces the number of Treg cells and downregulates IL-2 receptor (IL-2R) signaling and phosphorylation of STAT5, causing deficient SOCS1 suppression [29]. Yao et al. found that STAT3 and STAT5 phosphorylations are positively regulated by miR-155. That is probably due to miR-155 blocking the inhibitory effect on phosphorylations of STAT3 and STAT5 mediated by SOCS1 [10]. MiR-155 was assessed as a potential biomarker of response on methotrexate (MTX) in RA patients by AUC analysis by Singh et al. [30].

Our study has some limitations. The sample size should be enlarged. Further studies are necessary to elucidate the exact microRNAs influence on transcriptional factors as well as on Th17 and Treg cells in the etiology and pathogenesis of rheumatoid arthritis. Additional studies can identify and describe the microRNAs and their target genes signaling pathways, and interactions between them and Th17 and Treg cells included in the inflammatory processes, which may cause the development of new RA therapeutics.

## 4. Materials and Methods

### 4.1. Study Population

In this study, we used blood from 14 patients with RA and controls: 11 patients with OA and the group of 15 healthy controls (HCs), which were age and gender-matched to patients we used in this study. This study meets all criteria contained in the Declaration of Helsinki and was approved by the Ethics Committee of the National Institute of Geriatrics, Rheumatology, and Rehabilitation, Warsaw, Poland (approval protocol number 29 June 2016). All participants gave their written informed consent before enrollment. Patients with RA were recruited from the National Institute of Geriatrics, Rheumatology and Rehabilitation in Warsaw, Poland; and from the Poznan University of Medical Sciences, Poland. All RA patients fulfilled the American College of Rheumatology (ACR 2010) criteria for RA. Patients with OA were recruited from the National Institute of Geriatrics, Rheumatology and Rehabilitation in Warsaw, Poland. OA patients were diagnosed based on characteristic x-ray findings and the absence of features suggestive of inflammatory arthritis and must meet the ACR criteria for OA of the knee. RAand OA patients with an active infection, cancer, or other rheumatological diseases were excluded from the study. The HCs consisted of volunteers without clinical or laboratory signs of autoimmune diseases. The control group was chosen randomly between blood bank donors to match the age, gender, and ethnicity of patients with RA and OA. All participants who donated blood for this study had the same socioeconomic status and were from the same geographic region.

RA patients were qualified based on their physical examination and laboratory tests. The main factor based on which patients were qualified was age, gender, disease duration, swollen joints number, C-reactive protein (CRP), erythrocyte sedimentation ratio (ESR), platelets (PLT), and creatinine. Additionally, we evaluated them to study by presence of rheumatoid factor (RF ≥ 34 IU/mL), presence of anti-CCP antibodies (anticyclic citrullinated peptide autoantibodies, aCCP ≥ 17 IU/mL), disease activity score in 28 joints (DAS-28), visual analog scale (VAS, range 0–100), Larsen score, and information about the treatment were collected at the time of obtaining samples from patients. Demographic and clinical characteristics of subjects are summarized in Table 2 and detailed clinical characteristics of RA patients are summarized in Table 3.

### 4.2. Detection of Th17 and Treg Cells Using Flow Cytometry.

Using Ficoll-Paque (GE Healthcare Bio-Sciences, Uppsala, Sweden) and performing density gradient centrifugation, we were able to isolate PBMC. In the next step, cells were cultured in RPMI 1640 (Invitrogen, Paisley, UK), 10% heat-inactivated fetal bovine serum (FBS) (Gibco, Thermofisher, USA), 100 U/mL penicillin, and 100 ug/mL streptomycin (Sigma-Aldrich, Saint Louis, MO, USA) for 12 h. After that period, cells were harvested and stained for particular membrane antigens using anti-CD4 APC-Cy7, anti-CD25 PE, anti-CD127 FITC, anti-CCR6 APC, and anti-CXCr3 PE-Cy7 murine Abs. Cells were washed, then they were acquired, analyzed, and sorted using a FACSAria cell sorter/cytometer and Diva software. By using 7AAD staining, we were able to eliminate dead cells from analysis (Figure S3). All reagents used in the flow cytometry were purchased from Becton Dickinson (San Jose, CA, USA).

### 4.3. Isolation of Total RNA from Th17 and Treg Cells

Total RNA was isolated from earlier sorted Th17 and Treg cells using the miRNeasy Micro Kit (Qiagen, Germantown, MD, USA). The quantity and quality of isolated RNA were evaluated by the Quawell Q5000 spectrophotometer. Total RNA isolated during this procedure was used to perform the reverse transcription reaction using High-Capacity cDNA Reverse Transcription Kit (Applied Biosystems, Carlsbad, CA, USA). We stored synthesized cDNA at −20 °C for the next step. Preamplification of cDNA from this reverse transcription was performed using TaqMan^®^ PreAmp Master Mix Kit (Applied Biosystem, Carlsbad, CA, USA) according to the manufacturer instructions. The rest of the isolated total RNA from Th17 and Treg cells were used to perform reverse transcription reaction using the miRCURY LNA RT kit (Qiagen) according to the manufacturer instructions. Both reverse transcriptions were conducted in a thermocycler (SensoQuest Labcycler 48 s, Göttingen, Germany).

### 4.4. Quantitative Real-Time PCR

We used following TaqMan primer and probes (Applied Biosystems, Foster City, CA, USA) SMAD3 (Hs00969210_m1), STAT3 (Hs00374280_m1), STAT5a (Hs00559647_m1), SOCS1 (Hs00705164_m1), GAPDH (Hs02786624_g1), and RPLO (Hs99999902_m1). The following assays were used in microRNA experiment: hsa-miR-24-3p (QG-339306_YP00204260), hsa-miR-26a-5p (QG-339306_YP00206023), hsa-miR-31-5p (QG-339306_YP00204236), hsa-miR-100-5p (QG-339306_YP00205689), hsa-miR-126-3p (QG-339306_YP00204227), hsa-miR-146a-5p (QG-339306_YP00204688), hsa-miR-155-5p (QG-339306_YP00204308), hsa-miR326 (QG-339306_YP00204512), SNORD48 (hsa) (QG-339306_YP00203903), and U6 snRNA (has, mmu) (QG-33906_YP00203907). To prepare quantitative Real-Time PCR, we used TaqMan Gene Expression Master Mix (Applied Biosystems); and for quantitative with microRNA, we used miRCURY SYBR Green PCR Kit (Qiagen). Quantitative Real-Time PCR was performed on real-time cycler (Quant Studio 5, Applied Biosystem, Foster City, CA, USA). Each sample was analyzed in duplicates. From that, we took the mean Ct value, and we used it in the next steps of the analysis. Ct values higher than 35 were taken out of analysis and considered below quantification. The housekeeping gene has been selected and relative expression was calculated by ΔΔCt method or ΔCt method normalized to RPLO; in the case of microRNA, SNORD48 was used as a reference.

### 4.5. Statistical Analysis

For microRNA, we used the Shapiro–Wilk test as a normality test. Analysis of statistical signification between Th17 cells and Treg cells was conducted with the Wilcoxon test. Statistical significance between two independent groups was determined by the nonparametric Mann–Whitney U test. As for the comparison between RA, OA, and HCs, we used independent tests—the Kruskal–Wallis test with Dunn’s post hoc. Correlation analysis of the mRNA with microRNA in Th17 and Treg cells was conducted by the Spearman test. Data were analyzed using GraphPad Prism software version 8.2.1. and was presented as figures. In all tested values, p < 0.05 was considered significant. The receiver operating characteristic (ROC) curves analysis, an area under curves (AUC) likelihood ratio of chi-square, and p-value were obtained by multivariable logistic regression analysis and were calculated by the R program (R Development Core Team (2008) R: A language and environment for statistical computing. R Foundation for Statistical Computing, Vienna, Austria ISBN 3-900051-07-0, URL http://www.R-project.org.) with R:ggplot [31] and pROC [32] packages.

## 5. Conclusions

In conclusion, our association study revealed correlations of expression levels in Treg cells obtained from RA patients between miR-26 and SOCS1, miR-31 and SMAD3, miR-155 and SMAD3, SMAD4. Moreover, we also noticed correlations in Th17 cells from RA between miR-26 and SMAD3, STAT3, SOCS1, miR-155 and STAT3. The present study also showed that RA Treg cells have a negative correlation between STAT5a and miR-26 level as well as between miR-126 and STAT5a level. Our research established that miR-26 and miR-155 can be good possible biomarkers for rheumatoid arthritis and osteoarthritis. After analyzing gene expression in a tested group of patients, we assume that SMAD2 shows the most promise to be used in the future to allow for earlier recognition of RA. Our study presented some interesting observations about connections between microRNAs’ expression and the clinical characteristics of RA patients. The current study revealed that RA patients with DAS28 ≤ 5.1 have significantly higher miR-31 expression levels in Th17 cells and, on the contrary, the twice higher expression level of miR-24 in Treg cells from patients with DAS28 > 5.1. We also exposed that the expression level of miR-146a in Treg cells is much higher in RA patients with RF than those with RF-negative. Furthermore, our outcomes presented that anti-CCP positive RA patients have meaningfully lower miR-31 expression levels in Treg cells than anti-CCP negative RA patients.

We believe that our study may be useful in elucidating and describing the inflammatory processes leading to the RA development and RA course. There is a need for further studies with an enlarged sample size as well as the need for functional studies. Functional studies may provide insights into the effect of microRNAs on gene function. Further studies are essential to clarify the microRNAs’ impact on Treg and Th17 cells, as well as transcriptional factors in the development and the course of rheumatoid arthritis. Moreover, we trust that this knowledge can lead to the development of new therapeutics for rheumatoid arthritis.

## Figures and Tables

**Table 1:** Prognostic values of the relative expression level of the analyzed microRNAs in RA and OA patients as well as healthy subjects (HCs) based on the area under the curve receiver operating characteristic (ROC)–AUC (Area Under Curve).

microRNA	Group	AUC	Th17 *p*	Treg AUC	*p*
miR-26	HC RA	0.75	0.02	0.92	0.0002
	HC OA	0.66	0.16	0.86	0.0013
	RA OA	0.55	0.66	0.66	0.17
miR-155	HC RA	0.80	0.006	0.73	0.048
	HC OA	0.75	0.03	0.51	0.94
	RA OA	0.52	0.88	0.68	0.12

**Table 2:** Demographic and clinical characteristics of the study population. ESR—erythrocyte sedimentation ratio; CRP—C-reactive protein.

Parameter	RA n = 14	OA n = 11	HCs n = 15
Age	52 ± 19 64 (21–75)	70 ± 10 66.5 (56–85)	48 ± 6.6 45 (41–63)
Female/Male	13/1	7/4	9/6
ESR (mm/h)	14 (9–64)	15 (3–26)	
CRP (mg/L)	12.5 (5–73)	5 (5–10)	

**Table 3:** Detailed clinical characteristics of RA patients.

Clinical Characteristics of Patients with RA	
Disease duration (years)	6.5 (0.5–18)
DAS-28 > 5.1 n (%)	6 (42%)
Larsen 1–3 n (%)	9 (64%)
RF Positivity n (%)	5 (35%)
Anti-CCP Positivity n (%)	7 (50%)
Methotrexate (MTX) n (%)	9 (64%)
Somatostatin Analogue (SSA) n (%)	3 (21%)
Antimalarials Drug n (%)	7 (50%)
Glucocorticoids (GCs) n (%)	4 (28%)
IL-6 Inhibitor n (%)	1 (7.1%)
No Treatment n (%)	1 (7.1%)

## Supplementary Materials

The following are available online at https://www.mdpi.com/1422-0067/21/19/7169/s1.

## Abbreviations

aCCP	Anti-cyclin citrullinated peptide autoantibodies
AUC	Area under curve
CD	Cluster of differentiation
CNS1	Conserved noncoding sequence 1
CRP	C-reactive protein
DAS	Disease activity score
DNMT 1	DNA methyltransferase 1
ESR	Erythrocyte sedimentation ratio
FBS	Fetal bovine serum
FOXP3	Forkhead box P3
GCs	Glucocorticoids
HCs	Healthy controls
IL-2	Interleukin-2
Il-6	Interleukin-6
JAK/STAT	Janus kinase/signal transducers and activators of transcription
miRNAs, miRs	MicroRNAs
MS	Multiple sclerosis
MTX	Methotrexate
OA	Osteoarthritis
PBC	Primary biliary cholangitis
PBMCs	Peripheral blood mononuclear cells
PLT	Platelets
pTreg	Peripherally derived Treg
RA	Rheumatoid arthritis
RF	Rheumatoid factor
ROC	Receiver operating characteristic
SLE	Systemic lupus erythematosus
SMAD2	SMAD family member 2
SMAD3	SMAD family member 3
SMAD4	SMAD family member 4
SOCS1	Suppressor of cytokine signaling 1
SSA	Somatostatin analogue
STAT3	Signal transducer and activator of transcription 3
STAT5	Signal transducer and activator of transcription 5
Th cells	T helper cells, helper T cells
Treg cells	T regulatory cells, regulatory T cells
VAS	Visual analog scale.
