# Supplementary material for: The Interplay between Transcriptional Factors and MicroRNAs as an Important Factor for Th17/Treg Balance in RA Patients
Source: Int J Mol Sci. 2020 Sep 28;21(19):7169. doi: 10.3390/ijms21197169 (PMC7583886; doi:10.3390/ijms21197169)
Supplement: Supplementary file 1 [file ijms-21-07169-s001.zip › supplementary materials.docx]

*Supplementary Materials*

Table S1. Arrangement of selected microRNA in Treg and Th17 cells by diseases.

| **microRNA** | **Tested group** | **Treg** median (min-max) | **Th17** median (min-max) |
| --- | --- | --- | --- |
| **miR-24** | HC | 0.07006 (0.00135-0.9564) | 0.23353 (0.02853-2.23042) |
|  | OA | 0.0958 (0-0.36172) | 0.12952 (0-4.17253) |
|  | RA | 0.071295 (0-0.5258) | 0.098375 (0-0.34621) |
| **miR-26** | HC | 0.098375 (0-0.34621) | 0.06065 (0-0.20102) |
|  | OA | 0.02317 (0-0.11434) | 0.00537 (0-0.27039) |
|  | RA | 0.0053 (0-0.03223) | 0.00888 (0-0.02425) |
| **miR-31** | HC | 0.00325 (0-0.02934) | 0.00717 (0.00041-0.04177) |
|  | OA | 0.00173 (0-0.02182) | 0.00218 (0-0.05018) |
|  | RA | 0.002835 (0-0.02569) | 0.00465 (0-0.02042) |
| **miR-146a** | HC | 0.36432 (0-2.13913) | 0.08194 (0.0004-0.62698) |
|  | OA | 0.32016 (0-3.18028) | 0.075705 (0-5.3329) |
|  | RA | 0.27785 (0-3.63632) | 0.04206 (0.00299-0.19654) |
| **miR-155** | HC | 0.04713 (0.02211-0.79684) | 0.06181(0.00995-1.48942) |
|  | OA | 0.05155 (0-0.39726) | 0.01634 (0.00175-0.44475) |
|  | RA | 0.02911(0-0.13222) | 0.02271(0-0.06607) |

Fig. S1. Correlation between microRNAs in Treg cells in OA patients.

Table S2. Correlations between microRNAs in Th17 cells in OA patients.

| **microRNA** | **corellated**  **microRNA** | **correlation**  **coefficient** | | **p value** |  | |
| --- | --- | --- | --- | --- | --- | --- |
| **miR-24** | miR-146a | 0.70 | 0.0006 | | |  |
| **miR-26** | miR-146a | 0.83 | <0.0001 | | |  |
| **miR-26** | miR-155 | 0.89 | <0.0001 | | |  |
| **miR-31** | miR-155 | 0.70 | 0.007 | | |  |
| **miR-146a** | miR-155 | 0.73 | 0.004 | | |  |

**Table S3.** The cut-off points for expression levels of miR-26 and miR-155 in RA and OA patients and healthy subjects (HCs) with the determination of the level of sensitivity and specificity.

| **microRNA** | **Group** | **Cells** | **Cutoff value** | **Sensitivity % (95% CI)** | **Specificity % (95% CI)** | **LRT** |
| --- | --- | --- | --- | --- | --- | --- |
| **miR-26** | RA vs HC | Th17 | 0.022 | 93.33 (70.18-99.66) | 53.85 (29.14-76.79) | 2.02 |
|  |  | Treg | 0.025 | 84.62 (57.77-97.27) | 92.86 (68.53-99.63) | 11.85 |
|  | OA vs HC | Th17 | 0.020 | 76.92 (49.74-91.82) | 61.54 (35.52-82.29) | 2.00 |
|  |  | Treg | 0.051 | 84.62 (57.77-97.27) | 78.57 (52.41-92.43) | 3.90 |
| **mir-155** | RA vs HC | Th17 | 0.046 | 80 (54.81-92.95) | 61.54 (35.52-82.29) | 2.08 |
|  |  | Treg | 0.118 | 92.31 (66.69-99.61) | 30.77 (12.68-57.63) | 1.33 |
|  | OA vs HC | Th17 | 0.070 | 83.33 (55.20-97.04) | 46.16 (23.21-70.86) | 1.55 |
|  |  | Treg | 0.075 | 61.54 (35.52-82.29) | 30.77 (12.68-57.63) | 0.88 |


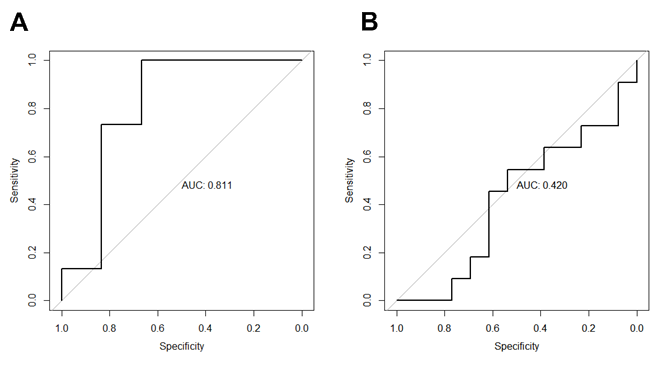


Fig. S2. ROC analysis. A- ROC analysis of the combination of miR-26 and miR-155 in Th17 RA vs HCs, B- ROC analysis for miR-146a in Treg OA vs HCs.


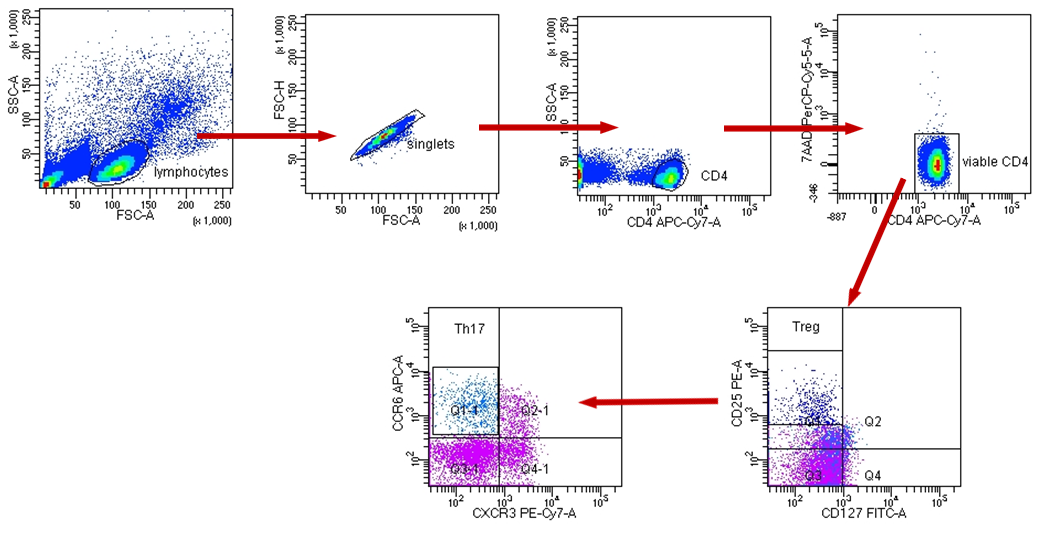


Figure S3. Representative gating strategy for FACS of PBMC, showing Treg and Th17 lymphocyte.
