# Supplementary material for: The Interplay between Transcriptional Factors and MicroRNAs as an Important Factor for Th17/Treg Balance in RA Patients
Source: Int J Mol Sci. 2020 Sep 28;21(19):7169. doi: 10.3390/ijms21197169 (PMC7583886; doi:10.3390/ijms21197169)
Supplement: Supplementary file 1 [file ijms-21-07169-s001.zip › supplementary materials.pdf]

Supplementary Materials

Table S1. Arrangement of selected microRNA in Treg and Th17 cells by diseases.

**microRNA**	**Tested group**	**Treg** median (min-max)	**Th17** median (min-max)
**miR-24**	HC	0.07006 (0.00135-0.9564)	0.23353 (0.02853-2.23042)
	OA	0.0958 (0-0.36172)	0.12952 (0-4.17253)
	RA	0.071295 (0-0.5258)	0.098375 (0-0.34621)
**miR-26**	HC	0.098375 (0-0.34621)	0.06065 (0-0.20102)
	OA	0.02317 (0-0.11434)	0.00537 (0-0.27039)
	RA	0.0053 (0-0.03223)	0.00888 (0-0.02425)
**miR-31**	HC	0.00325 (0-0.02934)	0.00717 (0.00041-0.04177)
	OA	0.00173 (0-0.02182)	0.00218 (0-0.05018)
	RA	0.002835 (0-0.02569)	0.00465 (0-0.02042)
**miR-146a**	HC	0.36432 (0-2.13913)	0.08194 (0.0004-0.62698)
	OA	0.32016 (0-3.18028)	0.075705 (0-5.3329)
	RA	0.27785 (0-3.63632)	0.04206 (0.00299-0.19654)
**miR-155**	HC	0.04713 (0.02211-0.79684)	0.06181(0.00995-1.48942)
	OA	0.05155 (0-0.39726)	0.01634 (0.00175-0.44475)
	RA	0.02911(0-0.13222)	0.02271(0-0.06607)

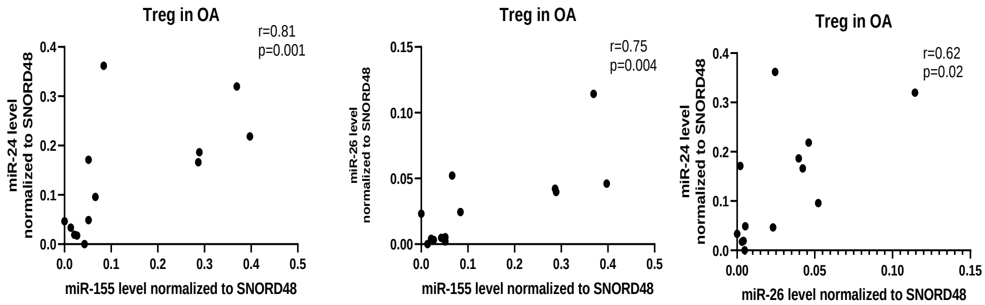

Fig. S1. Correlation between microRNAs in Treg cells in OA patients.

Table S2. Correlations between microRNAs in Th17 cells in OA patients.

microRNA	corellated microRNA	correlation coefficient	p value
**miR-24**	miR-146a	0.70	0.0006
**miR-26**	miR-146a	0.83	<0.0001
**miR-26**	miR-155	0.89	<0.0001
**miR-31**	miR-155	0.70	0.007
**miR-146a**	miR-155	0.73	0.004

Table S3. The cut-off points for expression levels of miR-26 and miR-155 in RA and OA patients and healthy subjects (HCs) with the determination of the level of sensitivity and specificity.

microRNA	Group	Cells	Cutoff value	Sensitivity % (95% CI)	Specificity % (95% CI)	LRT
**miR-26**	RA vs HC	Th17	0.022	93.33 (70.18-99.66)	53.85 (29.14-76.79)	2.02
		Treg	0.025	84.62 (57.77-97.27)	92.86 (68.53-99.63)	11.85
	OA vs HC	Th17	0.020	76.92 (49.74-91.82)	61.54 (35.52-82.29)	2.00
		Treg	0.051	84.62 (57.77-97.27)	78.57 (52.41-92.43)	3.90
**mir-155**	RA vs HC	Th17	0.046	80 (54.81-92.95)	61.54 (35.52-82.29)	2.08
		Treg	0.118	92.31 (66.69-99.61)	30.77 (12.68-57.63)	1.33
	OA vs HC	Th17	0.070	83.33 (55.20-97.04)	46.16 (23.21-70.86)	1.55
		Treg	0.075	61.54 (35.52-82.29)	30.77 (12.68-57.63)	0.88

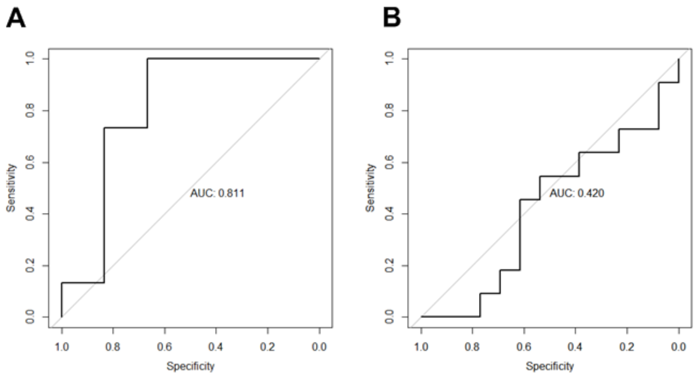

Fig. S2. ROC analysis. A- ROC analysis of the combination of miR-26 and miR-155 in Th17 RA vs HCs, B- ROC analysis for miR-146a in Treg OA vs HCs.

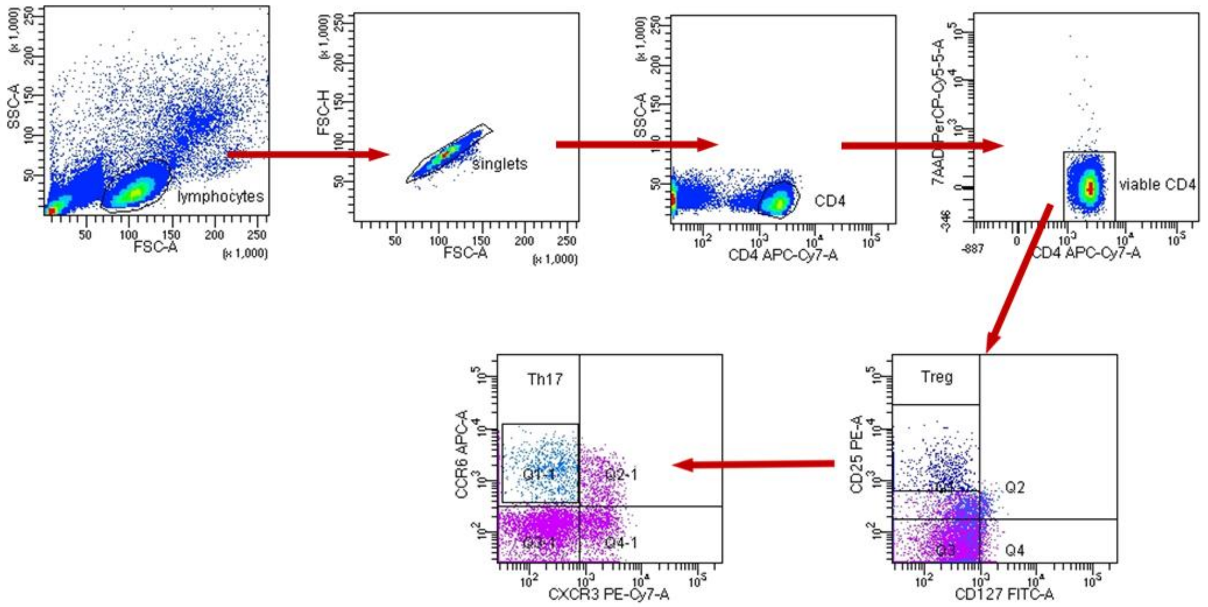

Figure S3. Representative gating strategy for FACS of PBMC, showing Treg and Th17 lymphocyte.
